# The complete mitochondrial genome and phylogenetic analysis of White-bellied Heron *Ardea insignis* (Pelecaniformes：Ardeidae)

**DOI:** 10.1080/23802359.2018.1532842

**Published:** 2018-10-26

**Authors:** Yubao Duan, Shimiao Shao, Yuan Li, Xu Luo

**Affiliations:** a Key Laboratory for Forest Resources Conservation and Utilization in the Southwest Mountains of China Ministry of Education, Southwest Forestry University, Kunming, Yunnan Province, China;; b College of Biodiversity Conservation and Utilization, Southwest Forestry University, Kunming, Yunnan Province, China

**Keywords:** *Ardea insignis*, complete mitogenome, phylogeny

## Abstract

The White-bellied Heron *Ardea insignis* is Critically Endangered wading bird. In this study, we first sequenced and described the complete mitochondrial genome and phylogeny of *A. insignis*. The whole genome of *A. insignis* was 18,656 bp in length and contained 14 protein-coding genes, 23 transfer RNA genes, two ribosome RNA genes, and two non-coding control regions. The overall base composition of the mitochondrial DNA was 30.86% for A, 24.60% for T, 30.48% for C, and 14.05% for G, with a GC content of 44.54%. A phylogenetic tree confirmed that *A. insignis* belonged to genus *Ardea*, family Ardeidae, and was sister to *A. purpurea*. This information will be useful in the current understanding of the phylogeny and evolution of Pelecaniformes.

The White-bellied Heron (*A. insignis* Hume, 1878) belongs to the family Ardeidae of order Pelecaniformes, is known from the eastern Himalayan foothills in Bhutan and north-east India to the hills of Bangladesh, north Myanmar and, historically at least, across west and central Myanmar (Gibb et al. 2013; BirdLife International 2018; Gill and Donsker 2018). This species is listed as of Critically Endangered (CE) on the IUCN Red List of Threatened Species (IUCN, 2018). The main threats are presumed to be widespread loss, degradation and disturbance of its habitats. At present, the complete mitochondrial genome of some Ardeidae has been studied (Zhang et al. 2012; Zhou et al. 2014; Tu et al. 2017). Due to the endangered degree of *Ardea insignis*, few study about *A. insignis* is reported. Therefore, we sequenced the complete mitochondrial genome of *A. insignis* to enhance our understanding of the phylogeny and evolution of Ardeidae.

The specimen was collected from Lushui Country located in the northwest of Yunnan Province in China (Han et al. 2015), and stored at College of Biodiversity Conservation and Utilization, Southwest Forestry University. The total mitochondrial DNA was extracted from the muscle tissue using Next-Generation Sequencing, and sequenced by using the Illumina Miseq Platform (Illumina, San Diego, CA). The complete mitochondrial genome of *A. insignis* was submitted to the NCBI database under the accession number MH737740. Phylogenetic tree of the relationships among Ardeidae were presented using 18 species by maximum parsimony analyses using PAUP* version 4.0b10 software with 1000 bootstrap replication (Swofford 2002). Bayesian inference was calculated with MrBayes3.1.2 with a general time reversible (GTR) model of DNA substitution and a gamma distribution rate variation across sites (Ronquist and Huelsenbeck 2003). Sequences of *Nipponia Nipponia* obtained from GenBank (KR862292.1) were used as outgroups to root trees following Zhou et al. (2014) and Huang et al. (2018).

The complete mitochondrial genome of *A. insignis* was 18,656 bp in length. A total of 41 mitochondrial genes were identified, including 14 protein-coding genes (PCGs), 23 transfer RNA (tRNA) genes, two ribosomal RNA (rRNA) genes, and two non-coding control region (D-loop). Among these genes, ND6 and nine tRNAs (*tRNA^Gln^*, *tRNA^Ala^*, *tRNA^Asn^*, *tRNA^Cys^*, *tRNA^Tyr^*, *tRNA^Ser^*, *tRNA^Glu^*, *tRNA^Pro^*, and *tRNA^Glu^*) were located on the light strand (Lstrand), while all of the remaining genes were located on the heavy strand (H-strand). The overall base composition of *A. insignis* mitogenome was 30.86% for A, 24.60% for T, 30.48% for C, and 14.06% for G. A + T content is 55.46%, which is higher than G + C content of 44.54%, similar to other Family Ardeidae (Gibb et al. 2006; Zhou et al. 2014).

The phylogenetic tree reconstructed from 14 PCGs of the *A. insignis* mitogenome (Figure 1). Our results supported the placement of *A. insignis* in Pelecaniformes (Genus *Ardea* Family Ardeidae) and confirmed that *A. insignis* was the sister lineage to *A. purpurea* with high support (99%) in Bayesian tree, similar to Chen et al. (2018). In all, the mitochondrial genome reported here would be useful in the current understanding of the phylogeny and evolution of Ardeidae.

**Figure 1.**
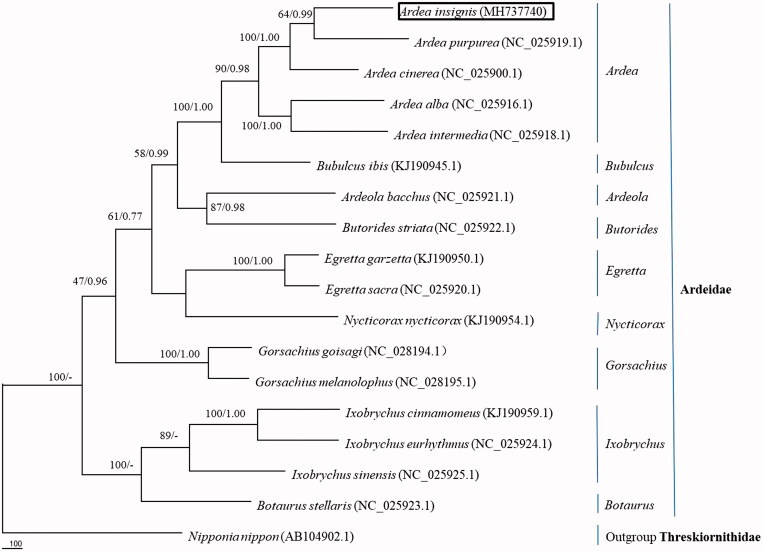
Phylogenetic tree of the relationships among Ardeidae. Branches received bootstrap support for maximum parsimony (BP, left) and Bayesian posterior probabilities (BPP, right).
